# Altered metabolic requirements in cancer cell migration and metastasis

**DOI:** 10.1186/1753-6561-6-S3-P32

**Published:** 2012-06-01

**Authors:** Jaewon Lee, M Rosa Ng, Kerstin W Sinkevicius, Carla F Kim, Gaudenz Danuser, Joan S Brugge, Marcia C Haigis

**Affiliations:** 1 Department of Cell Biology, Harvard Medical School, Boston, MA 02115, USA; 2 The Paul F. Glenn Labs for the Biological Mechanisms of Aging, Harvard Medical School, Boston, MA 02115, USA; 3 Stem Cell Program, Children’s Hospital Boston, Boston, MA 02115, USA; 4 Department of Genetics, Harvard Medical School, Boston, MA 02115, USA; 5 Harvard Stem Cell Institute, Cambridge, MA 02138, USA

## Background

Metastasis poses a great challenge in clinical management of many cancers. Metabolic demands of cancer cell proliferation – i.e. elevated aerobic glycolysis for biomass generation – have been well characterized, but the contribution of altered metabolism to metastasis remains to be elucidated. While elevated aerobic glycolysis, a phenomenon termed Warburg effect, is a hallmark of proliferative tumor cells, emerging evidence suggests that metastatic cancer cells have an opposite phenotype.

## Materials and methods

We investigated the metabolism in cell lines of different metastatic capacity. In addition, to quantitatively study cell migration and metabolism, we used MCF10A breast epithelial cells with fluorescent histone tags along with live cell imaging and tracking by a custom MATLAB program to measure speed and behavior of migration.

## Results

Our preliminary data suggest that the more metastatic cancer cells depend on mitochondrial metabolism. Furthermore, changing mitochondrial metabolism in MCF10A breast epithelial cell line affected not only the speed but also the pattern of cell migration. Consistent with the altered migratory behaviors, stimulation of mitochondrial metabolism changed cell adhesion markers.

## Conclusions

In sum, we show evidence that mitochondrial metabolism plays an important role in promoting cell migration and altering cell adhesion with implications for cancer metastasis.

